# Necrology

**Published:** 1895-08

**Authors:** E. C. Beckett


### NECROLOGY.

Dr. William Bryden, a well and favorably known veterinarian of Boston, died there on June 28th after an illness of about thirteen months, the immediate cause of death being apoplexy. Dr. Bryden was born in Scotland, having come to this country when a boy. He was graduated from the Montreal Veterinary College (now a department of the McGill University) in 1871, where for several years he served as one of its board of examiners. He was a past President of the United States Veterinary Medical Association; also a charter member and past President of the Massachusetts Veterinary Medical Association, and an honorary member when he died. For many years he was inspector of cattle for British steamships from the port of Boston; known personally to most members of the profession throughout the country, and to others by his frequent contributions to our journals.

He will be remembered by many young practitioners as a good friend and ever ready adviser. He was a good man whom we esteemed highly, of a genial and hospitable nature, of marked intelligence, an able student and practitioner.

Dr. Bryden was well up in Masonry, and will be greatly missed by a large circle of friends and acquaintances.

(E. C. Beckett, M.D.V.)

